# The Dutch Lower Extremity Functional Scale was highly reliable, valid and responsive in individuals with hip/knee osteoarthritis: a validation study

**DOI:** 10.1186/1471-2474-13-117

**Published:** 2012-07-02

**Authors:** Thomas J Hoogeboom, Rob A de Bie, Alfons A den Broeder, Cornelia HM van den Ende

**Affiliations:** 1 Department of Rheumatology, Sint Maartenskliniek, PO Box 9011, Nijmegen, 6500 GM, The Netherlands; 2 Caphri Research School, Maastricht University Medical Centre, Maastricht, The Netherlands; 3 Department of Epidemiology, Maastricht University Medical Centre, Maastricht, The Netherlands

## Abstract

**Background:**

The WOMAC is the most widely used self-report measure to evaluate physical functioning in hip or knee osteoarthritis, however its ability to discriminate pain and physical functioning (i.e. discriminate validity) has repeatedly been questioned. Little to no data is available on the discriminant validity of alternative questionnaires that measure the same construct, for instance the Hip and Knee Osteoarthritis Outcome Score (HOOS and KOOS, respectively) and the Lower Extremity Function Scale (LEFS). Therefore, we translated the LEFS to Dutch and studied its psychometric properties (i.e. validity, reliability and responsiveness). In addition, we assessed the discriminate validity of the LEFS, HOOS and KOOS.

**Methods:**

After translation with a forward/backward protocol, 401 individuals with hip or knee osteoarthritis completed the LEFS, HOOS/KOOS, SF-36, Hospital Anxiety and Depression Scale and Checklist Individual Strength questionnaires. To assess reliability and responsiveness, a sample of 106 and 108 patients completed a comparable set of questionnaires within 3 weeks and 3 months, respectively. Feasibility, validity, reliability and responsiveness were evaluated. Discriminant validity of the LEFS, HOOS and KOOS was examined by contrasting the scales’ correlations with the physical functioning subscale of the SF-36 with the scales’ correlations with the bodily pain subscale of the SF-36.

**Results:**

The Dutch version of the LEFS was feasible, had good internal consistency (0.96), good reliability (ICC = 0.86), good construct and discriminant validity, and showed no floor or ceiling effects. The minimal detectable change (MDC_90_) was ten points. Area under the receiver operating characteristic curve (AUC) analyses revealed good (AUC = 0.76) and fair (AUC = 0.63) responsiveness for the LEFS in improved and worsened patients, respectively. Discriminant validity for pain was apparent for the LEFS (*p* < 0.01), but not for the HOOS and KOOS (*p* = 0.21 and *p* = 0.20, respectively).

**Conclusions:**

Considering the LEFS’ good psychometric qualities and ability to discriminate between pain and functioning, we recommend the LEFS as the outcome measure of choice to assess self-reported physical functioning in individuals with hip or knee osteoarthritis.

## Background

Numerous self-report measures on physical function are available for the evaluation of patients with hip or knee osteoarthritis [1]. Among those, the licensed for use Western Ontario and McMaster University Osteoarthritis Index (WOMAC) [2] is the most widely used [3]. It is recommended by the Osteoarthritis Research Society for use in clinical trials in patients with hip or knee osteoarthritis to measure pain and disability [4]. However, consensus statements consistently advocate that pain and physical function must be measured independently [3,5]. A solid body of evidence demonstrates that the WOMAC-PF (Physical Function subscale) is unable to discriminate between pain and function [6-9].

Recently, three new license free self-report measures to determine functioning in patients with osteoarthritis have become available; the Hip Osteoarthritis Outcome Score (HOOS) [10], the Knee Osteoarthritis Outcome Score (KOOS) [11] and the Lower Extremity Function Scale (LEFS) [12]. One of those new measures, the LEFS, showed promise as a competitive alternative to the WOMAC-PF, as the LEFS can differentiate pain and functioning [13] and detect changes in functional status in the period immediately following surgery [14]. Moreover, the LEFS has excellent test-retest reliability, internal consistency and construct validity [12,13,15]. To date, it remains to be seen, whether the physical function scales of the HOOS and KOOS can discriminate between pain and physical function [10,11,16].

Since the LEFS is currently not available in Dutch, the primary purpose of this study was to evaluate the psychometric qualities of the Dutch LEFS in people with hip or knee osteoarthritis. Our secondary objective was to assess the discriminant validity for pain of the physical function subscale of the HOOS and KOOS and the LEFS.

## Methods

First the English version of the LEFS was translated into Dutch according to a standardized procedure described by Beaton *et al.*[17], and secondly it was tested for psychometric quality by use of prospective data.

### Procedure of translation

The translation procedure consisted of four steps. First, two persons translated independently of each other the English version of the LEFS into Dutch (forward translation) (T1 & T2); one translator (TJH) had a medical background and was familiar with the concepts of the questionnaire and the other (VvS) was a certified translator without a medical background. Both were native speakers. Based on a consensus meeting one final version (T-12) was formed. Second, two bilingual persons (T3 & T4) translated the T-12 questionnaire back into English (BT1 & BT2), to guarantee a consistent translation of the questionnaire. Both translators (PA & DKJ) were unfamiliar with the original questionnaire, the concepts of the questionnaire, and had no medical background. DKJ is also a certified translator. Third, an expert meeting was organised in which all translators, two health professionals (CKS, ML), a methodologist (CHMvE) and two language experts participated. During this meeting all versions of questionnaires (T1, T2, T-12, BT1, BT2) were combined and consensus on semantic, idiomatic, experiential and conceptual equivalence was reached resulting in a pre-final version of the questionnaire. The developers of the original questionnaire approved all previous steps and the final version. Finally, the pre-final version was presented in a group of 33 patients (20 women and 13 men; age (SD): 63 (13) years) to explore the clarity of the questionnaire. All patients were asked whether they understood the items and whether they could interpret the questionnaires correctly. Also, the time needed to complete the questionnaire was timed. The findings were discussed among the translators, resulting in only minor changes to the final Dutch version of the LEFS. Mean completion time was 3.5 (SD = 1.5) minutes. For the final version of the Dutch LEFS see Appendix 1.

### Patients and procedure

Individuals (≥18 years) diagnosed with hip or knee osteoarthritis (inclusion period June till October 2009) by an orthopaedic surgeon in the Sint Maartenskliniek hospital Nijmegen were eligible. People reporting concurrent rheumatoid arthritis, fibromyalgia or psoriatic arthritis, were excluded. Written materials were sent by mail: this included an information letter, an informed consent form, the questionnaires and a return envelope. At baseline, all patients completed four questionnaires, the LEFS, the HOOS or KOOS (depending on index joint), the SF-36 and the Hospital Anxiety and Depression Scale (HADS). A reminder was sent to those patients who did not respond within three weeks, to ensure a high response rate. One-hundred and twenty participants were sent a follow-up questionnaire to evaluate test-retest reliability (within 3 weeks) and another 120 participants were sent a follow-up questionnaire to evaluate responsiveness (after 3 months); as 100 participants were deemed sufficient [13]. By use of random numbers the 240 patients were selected to either the reliability or responsiveness study. Both follow-up mailings consisted of three questionnaires (LEFS, HOOS or KOOS, and the SF-36) and a global perceived effect question. For test-retest reliability, we considered a time interval of 3 weeks to be appropriate for the current population. For responsiveness, we deemed a period up to 3 months long enough to allow for improvement and brief enough to minimize the risk of a response shift [18,19].

The study was approved by the Institutional Review Board of the University Medical Centre Nijmegen (ID: 2009/20).

### Measures

The LEFS is a 20-item condition-specific questionnaire designed to be applicable to individuals with musculoskeletal conditions of the lower extremity [12]. Each item of the LEFS scores on a 5-point scale ranging from 0 to 4 points. When scoring the LEFS, up to 4 missing item responses are permitted, for more detailed information see Stratford et al. (2005) [20]. Accordingly, LEFS scores range from 0 to 80 points, with higher scores representing higher levels of functioning.

The HOOS and the KOOS include five subscales: Pain, other Symptoms, Function in Daily living (ADL), Function in Sport and Recreation (Sport/Rec), and hip/knee-related quality of life (QoL). Standardized response options are given (5-point Likert scale) and each question is scored from 0 to 4 points. Subsequently, a normalized score (100 indicating no symptoms and 0 indicating extreme symptoms) is calculated for each subscale. The Dutch HOOS and KOOS have good internal consistency, construct validity, no floor and ceiling effects and have been found to be reliable [10,11]. Both the HOOS and KOOS questionnaires include the WOMAC osteoarthritis-index in its complete and original format (with permission, http://www.koos.nu).

The SF-36 is a generic health status questionnaire which contains 36 items [21]. It measures eight major attributes (bodily pain; physical function; social function; role limitations because of physical problems; role limitations because of emotional problems; mental health; vitality; general health perceptions). It is widely used, reliable, validated into Dutch and is easy to complete. Higher scores indicate better health [22].

The Hospital Anxiety and Depression Scale (HADS) is a 14-item scale designed to detect anxiety and depression, independent of somatic symptoms [23]. It consists of two 7-item subscales measuring depression and anxiety on a 4-point response scale (from 0, no symptoms, to 3, maximum symptoms), with possible scores for each subscale ranging from 0 to 21. HADS is a valid and reliable screening instrument for detecting mood disorder in people with osteoarthritis [24,25]. Higher scores indicate higher levels of disorder.

Fatigue is measured with the 8-itemed “Subjective Fatigue” subscale of the Checklist Individual Strength (CIS) [26]. The outcomes per question are given in a 7-point scale, ranging from the statement ‘totally right’ to the statement ‘totally wrong’. The total score is counted in points with a range of 1-7 per question and a total score range of 8-56 points. The CIS is a sensitive instrument with good discriminating power and reliability [26].

The external criterion for distinguishing between improved and unimproved subjects was a 7-point global perceived effect (GPE) scale. The categories of improvement included the following: completely recovered, much improved, slightly improved, not changed, slightly worse, much worse, and vastly worsened.

### Statistical analyses

Descriptive statistics were used to describe the study population and the number of missing values. Data symmetry was tested by use of visual inspection of the data distribution plotted by histograms. Psychometric qualities of the LEFS were expressed by floor- and ceiling effects, internal consistency, test-retest reliability, minimally detectable change, construct validity, discriminant validity and responsiveness.

#### Floor and ceiling effects

Floor and ceiling effects were determined by calculating the number of individuals that obtained the lowest (0) or highest (80) scores possible and were considered present if more than 15% of the participants achieved the highest or lowest score [27].

#### Internal consistency and dimensionality

Internal consistency – an indicator for the homogeneity of a questionnaire - was assessed with Cronbach’s alpha and 95% confidence intervals (95% CI’s). Internal consistency is considered good when Cronbach’s alpha lies between 0.7 and 0.9 [28]. Dimensionality was assessed by performing principal component factor analysis with loading coefficient absolute value suppression at 0.40 on the LEFS, KOOS-PF and HOOS-PF to determine if the individual items loaded on a single factor. Factor extraction had three requirements: scree plot point of inflection at the second Eigenvalue, Eigenvalue cut-off >1.0, and ≥10% variance [29].

#### Reliability and minimal detectable change

Reliability concerns the degree to which the results of measurement are consistent across repeated measurements [28]. Test-retest reliability of the Dutch LEFS was determined by means of Intraclass Correlation Coefficients (ICCs) (two-way random effects model absolute agreement) and Bland and Altman plots [30]. The ICC(2,1) equals variance between patients divided by variance between patients plus variance between measurements plus error variance. The value of the ICC ranges from 0 to 1, where one represents perfect reliability of the measurement. Consequently, to quantify the reliability of the LEFS scores we determined the standard error of measurement (SEM = SD[√1-ICC]). The SEM is a representation of measurement error expressed in the same units as the original measurement. We quantified the minimal detectable change at the 90% and 95% confidence level (MDC_90_ and MDC_95_) by multiplying the point estimate of the SEM, the square root of 2 (to account for the error associated with repeated measurements), and the z score of 1.65 or 1.96 (resp. 90% or 95% confidence level); formula MDC_90_ = SEM * 1.65 * √2 and MDC_95_ = SEM * 1.96 * √2 [31].

#### Validity

Construct validity reflects the extent to which a particular measure consistently relates to other measures with theoretically derived hypotheses for the constructs that are being measured [28]. To evaluate the construct validity of the LEFS, we formulated a set of 16 hypotheses (eight for knee osteoarthritis and eight for hip osteoarthritis) about the expected magnitude and direction of relationships between the LEFS and other instruments. If 75% or more of the arbitrarily set number of 16 hypotheses were confirmed we defined the construct validity of the LEFS as good [32,33].

Discriminant validity was examined for the LEFS and the physical function subscale of the HOOS and KOOS, by contrasting its correlation with the PF subscale of the SF-36 with its correlation with the bodily pain subscale of the SF-36. Meng *et al*’s test for dependent data was used to evaluate the differences between those correlations [34].

#### Responsiveness

We studied the responsiveness of the LEFS and the WOMAC-PF extracted from the HOOS-PF and KOOS-PF) in a combined hip and knee group, as only a very small number of patients reported clinically important change, thus not allowing to study the responsiveness of the HOOS and KOOS separately. As yet, a variety of responsiveness statistics is available. However, it is not yet known which of these statistics is better for assessing responsiveness [35] we utilized three different analyses. First we determined the Responsiveness Ratio of Guyatt (GRI: average change of recovered patients (GPE = 1-2)/SD of average change of stable patients (GPE = 3-5)). If the responsiveness ratio is larger than 1, the mean change score in clinically improved patients exceeds the measurement error and the instrument may be considered to be responsive, to an extent that is proportional to the magnitude of the responsiveness ratio [36,37]. Second, we determined the Standardized Response Mean (SRM: average score change/SD of score change). By use of the modified Jackknife testing, we assessed differences in SRM statistically [38]. Third, we calculated Receiver operating characteristic curves (ROC) for the improved subjects and for the worsened subjects using the change scores of the questionnaires and the patients’ ratings of change [39]. The patients’ rating of change was dichotomized to identify those subjects who experienced a clinically meaningful reduction of symptoms. Important change was defined as ‘Much Improvement (GPE = 1-2)’ or ‘Much Decline (GPE = 6-7)’. Consequently, we computed the area under the curve (AUC). An AUC of 1.0 indicates perfect discrimination, whereas an AUC of 0.50 indicates no performance better than chance.

## Results

Four-hundred and one individuals returned the baseline questionnaire in the study (response rate 82%). After the baseline questionnaire, 121 participants received a follow-up mailing to evaluate test-retest reliability (106 responded (88%)) and 125 participants received a follow-up mailing to evaluate the responsiveness (112 responded (90%)). Patient characteristics at baseline and follow-up are presented in Table 1.

**Table 1:** Patient and disease characteristics at baseline from the total group and the follow-up data from the reliability and the responsiveness sample*

***N***	**Baseline sample**		**Reliability sample** (≤3 weeks)		**Responsiveness sample** (≥3 months)	
	**401**		**106**		**112**	
**Age**, mean (SD)	58 (13)		61 (11)		58 (11)	
**Female**, ♀%	231 (58%)		65 (63%)		59 (54%)	
**Index joint**, knee	284 (71%)		81 (76%)		81 (72%)	
**BMI**, median (IQR)	26 (24-29)		26 (24-30)		27 (24-30)	
**Education**, higher^†^	77 (20%)		25 (25%)		27 (25%)	
**Duration complaints**, >5 years^†^	144 (61%)		72 (63%)		59 (58%)	
**Co-morbidities**, yes	192 (52%)		52 (50%)		63 (72%)	
	**Baseline data**		**Follow-up data**			
	**Hip group**	**Knee Group**	**Hip group**	**Knee Group**	**Hip group**	**Knee Group**
***N***	**117**	**284**	**25**	**81**	**31**	**81**
**LEFS**	36.0 (16.6)	39.6 (14.1)	29.8 (13.5)	36.4 (13.7)	34.3 (17.0)	37.5 (15.0)
**HOOS/KOOS**						
Pain, mean (SD)	44.6 (21.3)	49.9 (20.0)	41.7 (21.1)	46.5 (19.7)	43.6 (23.7)	53.8 (19.3)
Symptoms, mean (SD)	41.6 (21.4)	54.6 (19.3)	40.2 (20.3)	52.5 (20.5)	39.7 (23.5)	55.8 (19.6)
Function, daily living, mean (SD)	47.5 (21.9)	56.5 (21.7)	43.8 (21.0)	54.2 (20.3)	46.5 (24.0)	57.8 (21.4)
Function, sports and recreation, median (IQR)	25 (6-38)	10 (0-30)	13 (6-25)	5 (0-25)	19 (6-31)	10 (0-25)
Quality of life, median (IQR)	25 (13-38)	25 (13-44)	31 (19-38)	25 (13-38)	25 (13-38)	31 (19-44)
**SF36**						
Pain, mean (SD)	35.6 (8.9)	37.3 (8.2)	35.6 (6.5)	38.6 (7.3)	37.0 (8.0)	39.9 (8.3)
Functioning, mean (SD)	31.8 (9.8)	32.8 (9.2)	29.8 (8.5)	32.5 (8.2)	31.0 (10.0)	33.4 (9.5)
**HADS (total)**, median (IQR)	9 (5-16)	9 (4-15)	N/A	N/A	N/A	N/A
**CIS**, mean (SD)	34.8 (12.6)	31.0 (13.6)	N/A	N/A	N/A	N/A

* Figures are numbers and percentages in the brackets unless indicated differently. ^†^Cut-off arbitrarily chosen. Abbreviations: BMI = Body Mass Index, IQR = Inter Quartile Range, SD = Standard Deviation.

The majority of patients (86%) had less than three missing values. The proportion of missing values in the LEFS questionnaire (4%) was slightly less than the proportion of missing values in the KOOS (5%) and the HOOS (8%) questionnaires. The item ‘getting in or out of bath’ had the highest number of missing values in each of the questionnaires; 5% in the HOOS, 7% in the LEFS and 10% in the KOOS.

### Floor and ceiling effects

None of the 401 participants reported the lowest possible score whereas one patient (0.26%) reported the highest functional level implying that the Dutch LEFS has no floor or ceiling effects. In addition, the distribution of the LEFS was symmetrical.

### Internal validity and factoral structure

The internal consistency for the total group of patients (n = 401) reached a Cronbach’s alpha of 0.96 (lower limit (LL) 95%-CI: 0.95) for the 20 items. For the hip and knee osteoarthritis group Cronbach’s alpha reached 0.97 (LL 95%-CI: 0.96) and 0.95 (LL 95%-CI: 0.94), respectively. Within-scale principal component factor analysis revealed that all items included in the LEFS, KOOS-PF and HOOS-PF loaded on a single major factor (Table 2).

**Table 2:** Factor Analysis: Variance Explained for the LEFS, KOOS and HOOS*

**Factor**	**Initial Eigenvalues**		
	**Total**	**% of variance**	**Cumulative %**
**LEFS**			
Factor 1	10.84	54.18	54.18
Factor 2	1.87	9.33	63.51
Factor 3-20	0.96 - 0.06	36.49	100.00
**KOOS**			
Factor 1	9.76	57.42	57.42
Factor 2	1.46	8.61	66.03
Factor 3-17	0.86 - 0.05	33.97	100.00
**HOOS**			
Factor 1	9.69	57.01	57.01
Factor 2	1.50	8.83	65.84
Factor 3	1.02	5.98	71.82
Factor 4	0.94 - 0.07	28.18	100.00

* A principal component factor analysis with loading coefficient absolute value suppression at 0.40.

### Reliability and minimal detectable change

Within three weeks after the baseline questionnaire, five individuals improved (5%) (GPE = 1-2), three worsened (3%) (GPE = 6-7) and the majority (92%) remained stable (GPE = 3-5). Two-way random effects ANOVA demonstrated that the ICC of the Dutch LEFS questionnaire for the total group (n = 106) was 0.86. For the knee group (n = 81) and the hip group (n = 25) the ICC was 0.87 and 0.78, respectively. The standard error of measurement was 4.4 points. The MDC_90_ and MDC_95_ of the LEFS questionnaire was 10 points and 12 points, respectively.

**Figure 1:**
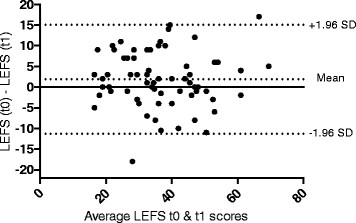
Bland & Altman plot.

The Bland-Altman plot (Figure 1) shows that the mean difference between the two applications of the LEFS was 1.87 points (95%-CI 0.22 to 3.52). The limits of agreement (mean ± 1.96 SD) ranged from -11.56 to 15.30 points.

### Validity

Thirteen of the 16 predefined hypotheses to determine the construct validity were confirmed (81%) (Tables 3 and 4). The following three hypotheses could not be confirmed. In the hip group we found a correlation of 0.55 between LEFS and CIS scores, which was higher than the predefined cut-off of 0.5. In the knee group we found that the duration of complaints did not influence the LEFS scores and that education level (primary, secondary or higher education) did influence the LEFS scores.

**Table 3:** Predefined hypotheses and the confirmation or rejection of the hypotheses for hip OA (n = 117) and knee OA (n = 284)

	**Hip OA**	**Knee OA**
There is a moderate correlation (*r* > 0.6) between LEFS scores and HOOS/KOOS-PF subscale.	**Yes**	**Yes**
The correlation between the LEFS and the HOOS/KOOS-PF subscale is higher than the correlation between the LEFS and the other subscales of the HOOS/KOOS.	**Yes**	**Yes**
The correlation between LEFS and HADS scores is low (*r* < 0.5).	**Yes**	**Yes**
The correlation between LEFS and CIS scores is low (r < 0.5).	No	**Yes**
Patients with multiple painful lower limb joints demonstrate lower LEFS scores than patients with pain in a single joint.	**Yes**	**Yes**
Patients with complaints less than 5 years demonstrate higher LEFS scores than patients with complaints over 5 years.	**Yes**	No
The participants’ education level (primary, secondary or higher education) does not influence the LEFS scores.	**Yes**	No
Sociodemographic characteristics, such as sex, doing volunteer work, income, type of health insurance, and family status, are unrelated (r < 0.2) to the LEFS score:	**Yes**	**Yes**
	7/8 (88%)	6/8 (75%)

Abbreviations: CIS = Checklist Individuals Strength, HADS = Hospital Anxiety and Depression Questionnaire, HOOS = Hip Osteoarthritis and Outcome Score, KOOS = Knee Osteoarthritis and Outcome Score, LEFS = Lower Extremity Functional Scale, OA = osteoarthritis, PF = physical function.

**Table 4:** Correlation data for the hip OA (n = 117) and knee OA (n = 284) groups at baseline

	**Correlation with LEFS**	
	**Hip group**	**Knee group**
	*r* (95%-CI)	*r* (95%-CI)
**HOOS/KOOS**		
Pain	0.71 (0.60 – 0.79)	0.65 (0.58 - 0.72)
Symptoms	0.56 (0.41 – 0.67)	0.42 (0.32 – 0.52)
Function, daily living	0.78 (0.69 – 0.84)	0.78 (0.73 – 0.83)
Function, sports and recreation	0.74 (0.64 – 0.82)	0.63 (0.55 – 0.70)
Quality of life	0.72 (0.62 – 0.80)	0.60 (0.52 – 0.67)
**SF36**		
Pain	0.51 (0.35 – 0.64)	0.66 (0.59 – 0.72)
Functioning	0.82 (0.75 – 0.88)	0.81 (0.77 – 0.85)
**HADS**		
** Anxiety**	−0.25^†^ (-0.42 – -0.07)	−0.10 (-0.22 – 0.02)
** Depression**	−0.30^†^ (-0.46 – -0.12)	−0.28 (-0.38 – -0.16)
** CIS**	−0.55^†^ (-0.67 – -0.41)	−0.47 (-0.56 – -0.37)

^†^Due to the inverted scales, the correct interpretation of negative associations is that more anxiety/depression/fatigue is associated with worse function.

Abbreviations: CIS = Checklist Individuals Strength, HADS = Hospital Anxiety and Depression Questionnaire, HOOS = Hip Osteoarthritis and Outcome Score, IQR = Inter-quartile range, KOOS = Knee Osteoarthritis and Outcome Score, LEFS = Lower Extremity Functional Scale, OA = osteoarthritis.

Meng *et al*’s test demonstrated that the association of the LEFS with the SF-36 subscale pain differed significantly with the SF-36 subscale physical functioning (Table 4), indicating that the LEFS has discriminant validity for pain (*p* < 0.01). We found no significant differences between the association with SF-36 subscale’s pain and physical functioning and the HOOS-PF (r (95%-CI) = 0.64 (0.51 - 0.74) and 0.71 (0.60 - 0.79), p = 0.21) and the KOOS-PF (0.69 (0.62 - 0.75) and 0.73 (0.67 - 0.79), *p* = 0.20, respectively), indicating that both questionnaires do not discriminate between pain and physical functioning.

### Responsiveness

Seven people (7%) reported relevant improvements in function (GPE = 1-2), nine people reported relevant worsening (8%) (GPE = 6-7) and the majority remained stable (85%) (GPE = 3-5). Responsiveness Ratio of the LEFS was 1.49, close to the outcomes of WOMAC-PF (1.20) and SF36-PF (1.22) (Table 5). Modified Jackknife testing demonstrated no statistical differences between the SRM for the LEFS (0.13) compared with the SRM of the WOMAC (SRM = 0.02, p = 0.45) and SF-36 (SRM = 0.00, p = 0.36). ROC curve analysis revealed that for improved patients the AUC was 0.76 (95% CI: 0.49 - 1.00) for the LEFS, 0.71 (95% CI: 0.45 - 0.98) for the WOMAC-PF (extracted from the HOOS-PF and KOOS-PF) and 0.68 (0.44 - 0.93) for the SF36-PF. For worsened patients the AUC was 0.63 (95% CI: 0.42 - 0.83) for the LEFS, 0.56 (0.34 - 0.78) for the WOMAC-PF and 0.56 (0.35 - 0.78) for the SF36-PF.

**Table 5:** Responsiveness ratio (GRI)

	Mean change	SD (baseline)	SD (change)	Mean change (GPE = 1-2)	SD mean change (GPE = 3-5)	SRM	GRI
LEFS	1.42	14.41	11.25	12.50	8.38	0.13	1.49
WOMAC-PF	0.19	14.84	8.86	9.66	8.06	0.02	1.20
SF36-PF	0.00	22.73	13.68	14.44	11.86	0.00	1.22

Abbreviations: GPE = Global perceived effect, GRI = Guyatt Responder Ratio, LEFS = Lower Extremity Functional Scale, SD = Standard Deviation, SF-36-PF = Short Form 36 - Physical Function scale, SRM = Standardized Response Mean, WOMAC = Western Ontario and McMasters University Osteoarthritis Index - Physical Function scale.

## Discussion

The primary objective of this study was to create a reliable and valid Dutch version of LEFS by translation and adaptation. No difficulties were encountered in the translation phase of the study; the structure of the original LEFS was not altered and all items were maintained. Moreover, participants reported no problems in the administration of the questionnaire. Considering the results of this validation study, we deemed the Dutch version of the LEFS to be an internally consistent, uni-dimesional, highly reliable and valid questionnaire to determine lower extremity functioning in patients with hip or knee osteoarthritis. Finally, the LEFS revealed good responsiveness by detecting improvement in patient GPE; however this finding should be interpreted with caution, given the small proportion of patient to actually report clinically relevant functional improvement. For our secondary objective, we were unable to demonstrate that the HOOS-PF and KOOS-PF subscales are able to discriminate between pain and physical function.

Construct validity of the Dutch version of the LEFS was good as most of the pre-formulated hypotheses were met. Three of the 16 hypotheses could however not be confirmed. First, in the hip group, the correlation between the lower extremity functioning (LEFS) and fatigue (CIS) was over 0.5 in the hip group, however similar correlations were found for HOOS-PF (r = 0.55) and SF-36 PF (r = 0.50). As comparative measures also demonstrate such a relation, fatigue might have a stronger relation with functioning than previously thought [10,15]. An important difference with previous studies is that we investigated fatigue with a fatigue-specific questionnaire in contrast to others that used the vitality scale of the SF-36 [10,15]. Second, participants with knee symptoms for less than five years did not report significantly less symptoms than patients with symptoms for over 5 years. Again this finding was also found for the KOOS-PF (*p* = 0.90) and the SF-36 PF (*p* = 0.75). These findings, could however, be biased by a phenomenon called response shift, which could have resulted in an underreporting of functional disabilities in the group with the longest duration of complaints [40]. Third and final, in the knee group we found that participants’ education level (primary, secondary or higher education) did influence the LEFS scores. It would be undesirable if LEFS scores were influenced by education level, as this would indicate that the LEFS is difficult to interpret. Further scrutiny of this finding indicates that patients with knee symptoms who enjoyed a higher education reported less symptoms than patients without or only primary education (*p* = 0.02); also when adjusted for age, sex, BMI, co-morbidities, duration of complaints and being employed. Yet again, this finding was also found for the KOOS-PF (*p* = 0.04), but not for the SF-36 PF subscale (p = 0.08). Our findings are in contrast to a previous study that addressed the relation between the LEFS scores (Italian version) and education levels. This discrepancy can possibly explained by the different format of the Italian version; an interview-format instead of a self-reported questionnaire [15]. It would be of interest to further elucidate this relation in other studies.

Although the responsiveness of the Dutch LEFS was good and superior to the WOMAC-PF and SF36-PF, compared to Italian validation study by Cacchio *et al.* (2010) (AUC = 0.86) it was somewhat low [15]. On the other hand, the psychometric properties of the Dutch LEFS (i.e. Cronbach’s alpha [12,15], reproducibility [12,13,15] and validity [12,13]) were comparable to the findings of previous validation studies. Our results regarding the responsiveness of the LEFS, WOMAC-PF and SF36-PF, should be interpreted with caution. Given the small number of patients reporting clinically relevant change which may have impacted for example the magnitude of the SRM, the point estimates might be spurious. Future (intervention) studies should further investigate the responsiveness of the Dutch LEFS.

The lack of discriminant validity for the WOMAC-PF has been demonstrated in numerous occasions [6-9,41,42]. Therefore, the greater discriminant validity of the LEFS compared to the WOMAC-PF [13,14] was one of the foremost reasons to translate and adapt the LEFS to the Dutch language. In our study we compared the LEFS questionnaire to the HOOS-PF and KOOS-PF subscales. As the physical function subscale of the HOOS and KOOS are very similar to the WOMAC-PF, these subscales are also at great risk for lacking discriminant validity. Our results indicate that the LEFS, but not the KOOS-PF and the HOOS-PF, could discriminate from pain measures, that is, KOOS-PF and HOOS-PF did not show a statistically higher correlation with the PF subscale than with the bodily pain subscale of the SF-36, whereas the LEFS did. As far as we know, we are the first to also demonstrate the lack of discriminant validity in the (Dutch version of the) HOOS and KOOS subscales, as in those particular validation studies only SF-36 subscales other than the bodily pain subscale were examined [10,11,16].

A limitation of our study is that we recruited only individuals with hip and knee osteoarthritis. Originally the LEFS has been developed as a measure that could be used for all kinds of conditions of the lower extremity [12]. The exclusion of other condition hampers the generalizability of our findings to other complaints of the lower extremity. We did however evaluate the LEFS ability to differentiate between patients with and without additional lower extremity pain co-morbidities, which demonstrated a linear association between the number of lower extremity joint pain co-morbidities and LEFS scores. The latter analysis showed promise that the Dutch version of the LEFS is also able to detect functional disabilities in patients with other symptoms than just hip and knee osteoarthritis. Another limitation of this study is that we did not assess the association between the Dutch version of the LEFS and a set of performance measures to determine the convergent validity. Future studies should investigate this association. A third limitation, the Cronbach’s Alpha value surpassed the cut-off value of 0.90 indicating item redundancy. However, due to the magnitude of our study sample and relatively high number of items this figure might have been inflated [43]. Finally, we studied the construct validity of the LEFS by testing hypotheses according to prespecified cut-off values; however cut-off value are often too rigid by their dichotomous (true/false) nature. Future studies should consider using the lower or upper bound of the 95% confidence interval of an association.

## Conclusions

We found that the Dutch version of the LEFS has no floor and ceiling effects, good internal consistency, reliability, construct validity and responsiveness. Moreover, the Dutch LEFS demonstrated discriminant validity for pain, as it was able to discriminate between pain and physical functioning, whereas both the HOOS-PF and KOOS-PF did not. Therefore, we recommend the use of the Dutch LEFS as an outcome measure for physical functioning in patients with hip and/or knee osteoarthritis.

## Appendix A. Dutch version of LEFS

Beste meneer/mevrouw,Heeft u of zou u vandaag enige moeite hebben met de volgende bezigheden?Vult u alstublieft alle items in, ook wanneer u de bezigheden niet meer doet.Score: _____/80 punten.

## Competing interests

The authors declare that they have no competing interests.

## Authors’ contributions

Authors TJH, RAdB, AAdB, CHMvdE; 1) have all contributed to conception and design of this study; 2) have been involved in drafting the manuscript and revising it critically for important intellectual content; and 3) have given final approval of this version to be published.

## Pre-publication history

The pre-publication history for this paper can be accessed here:

http://www.biomedcentral.com/1471-2474/13/117/prepub
